# Continued Metacarpal Cortical Bone Growth in Mid to Late Adolescence: A Longitudinal Study of Cortical Bone Acquisition in a Documented Sample of 16‐ to 20‐Year‐Olds

**DOI:** 10.1002/ajpa.70186

**Published:** 2025-12-18

**Authors:** Maris A. Schneider, Rebecca J. Gilmour

**Affiliations:** ^1^ Department of Sociology & Anthropology Mount Royal University Calgary Alberta Canada; ^2^ Department of Anthropology Western University London Ontario Canada

**Keywords:** bone apposition, epiphyseal fusion, peak bone mass, periosteal envelope, radiogrammetry

## Abstract

**Objectives:**

Metacarpal radiogrammetry is widely used by anthropologists and archaeologists to assess cortical bone loss. However, the dynamics of metacarpal cortical bone acquisition, as it relates to epiphyseal fusion during late adolescence, requires greater clarification. This research uses the Burlington Growth Study, a longitudinal dataset of digitized hand‐wrist radiographs from a known age and sex sample, to investigate cortical bone growth in the second (MC2) and third (MC3) metacarpals of adolescents aged 16–20 years old.

**Materials and Methods:**

Medullary widths (MW), total widths (TW), and cortical indices (CI) of fully fused MC2 and MC3 bones were measured on digitized radiographs for 54 individuals (28 females, 26 males). Cortical bone thickness changes at the periosteal and endosteal margins were quantified and compared over the four‐year period from ages 16–20.

**Results:**

CI increased in the MC2 and MC3 of both sexes, indicating continued bone apposition after fusion. With the exception of female MC2s, this change is predominantly located at the periosteal surface (marked by an increasing TW). Cortex growth slows around 18 years old, with no significant changes in CI, TW, or MW between the ages of 18 and 20.

**Discussion:**

In the MC2 and MC3, cortical bone continues to grow in thickness in the years immediately following epiphyseal fusion. Skeletal maturity of these elements, as represented by fusion, does not equate to the cessation of cortical apposition. These findings contribute insight into bone development at the adolescent‐adult age transition and specifically serve to refine radiogrammetry age‐based inclusion or categorization criteria.

## Graphical abstract

Cortical bone of the second and third metacarpals continues to grow in thickness in the years immediately following epiphyseal fusion.

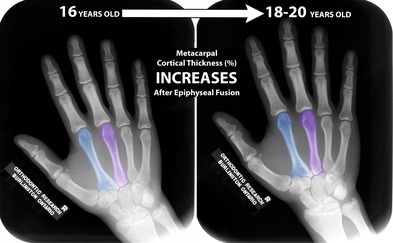

## 1 Introduction

Metacarpal radiogrammetry emerged as a useful clinical technique due to its ability to detect early bone loss in a non‐invasive and cost‐effective way. The method was first applied to evaluate cortical thickness and bone mineral density relationships among the metacarpal, spine, and femur (Barnett and Nordin 1960; Virtama and Mähönen 1960). Meema and Meema (1987) later refined a clinically standardized version of the technique for assessing bone loss and its relationship to osteoporotic fracture risk in menopausal women. Since then, metacarpal radiogrammetry has played a role in reducing the risk of future osteoporosis‐related fractures by enabling early detection, intervention, and treatment (Wilczek et al. 2013). In addition to investigations of age‐related bone loss in women, researchers have broadly applied metacarpal radiogrammetry to examine cortical bone loss in men (Wilczek et al. 2013), the influence of economic disparity and food resource access on bone development (Magan et al. 2017), the impact of physical activity on bone development and loss (Warden et al. 2014), and the effects of lifestyle (e.g., smoking, alcohol consumption, education, and body mass index) on cortical bone development and loss (Haara et al. 2006). Due to its clinical background, ease of application, and the availability of modern comparative datasets, metacarpal radiogrammetry has also been adopted to study adult age‐related cortical bone loss and osteoporosis trends in archaeological populations (Curate et al. 2019; Glencross and Agarwal 2011), along with other factors such as occupation (Mays 2001), handedness (Ubelaker and Zarenko 2012), and diet (Pfeiffer and King 1983).

To identify bone loss in adults, radiogrammetric approaches often compare measured cortical bone characteristics (e.g., total diaphyseal width, medullary width, cortical thickness) with those of individuals at their “peak bone mass.” Peak bone mass is the point at which skeletal mass is greatest over the life course and, on average, is typically achieved by 18.8 years in females and 20.5 years in males (Baxter‐Jones et al. 2011). Comparing individuals at an age of increased risk of bone loss to those at an age of peak bone mass allows for the magnitude of bone loss and the risk for fragility‐associated fractures to be more clearly identified (e.g., Curate et al. 2019; Haara et al. 2006; Ives and Brickley 2005; Robb et al. 2012). In archaeological metacarpal radiogrammetry studies, the youngest age included in this comparative peak bone mass category can vary from the point of fusion (at approximately 14.5–16.5 years old, according to Scheuer and Black 2000) to 20 years old (Beauchesne and Agarwal 2014; Gilmour et al. 2021; Glencross and Agarwal 2011; Ives and Brickley 2005; Robb et al. 2012; Mays 2011; Umbelino et al. 2019). This young adult category is then frequently capped at approximately 29–30 years of age, and sometimes as old as 35 years (Wesp and Hernández López 2022; Lazenby 1998). In cases where the youngest fully fused individuals are included, this decision is likely justified by the assumption that bone growth is essentially complete upon fusion and the rationale that these individuals might help maximize sample sizes, especially in instances where preservation is poor.

In an effort to clarify the lower age boundary of the comparative peak bone mass category, this study investigates whether skeletal maturity, as defined by epiphyseal fusion, truly signifies the cessation of bone growth or if cortical bone continues to accrue after fusion. While the fusion of epiphyses marks the end of longitudinal growth, animal models show that skeletal elements continue to grow via apposition in the years following epiphyseal fusion (Ravanetti et al. 2015; Yılmaz et al. 2023a; Yılmaz et al. 2023b). Studies on rabbits examined bone mineral apposition in the femur and tibia and reported a continuation of cortical bone apposition and consistent bone modeling rates following fusion (Cacchioli et al. 2012; Ravanetti et al. 2015). Similarly, Rogers et al. (2021) reported that horses typically reached 98% of their mature height by 24 months (equivalent to the human post‐pubertal age), and that moderate appositional bone growth continued to occur in their axial skeleton following this period. While animal models demonstrate this continued cortical apposition, they are limited as human proxies due to their inability to comprehensively reflect humans' prolonged adolescent growth periods that include periods of rapid growth spurts (Baxter‐Jones et al. 2011; Whiting et al. 2004).

In addition to animal models, researchers have also examined appositional bone changes after epiphyseal fusion across various human skeletal elements, recognizing that different elements reach peak bone mass at different average ages. For example, in humans, peak bone mineral content in the lumbar vertebrae is typically achieved between the ages of 22 and 28, and a little earlier in the distal radius, between 21 and 23 years old (Henry et al. 2004). A study by Parfitt et al. (2000) on growing ilia calculated that after epiphyseal closure, these elements continue to grow appositionally until at least 20–23 years of age. Kurki et al. (2022) investigated femoral and humeral cortical bone shape and area among growing individuals until approximately 23 years old to characterize the developmental trajectories of these bone cross‐sectional parameters. Although establishing continued apposition was not a direct aim of their study, their bivariate plots of percent cortical area compared to age at death suggest that percent cortical area in humeral and femoral diaphyses may continue to increase until approximately 23 years of age (the maximum age included in their study). Considering the typical age of fusion completion for each element (Humerus: 14–19 years [female], 16–21 years [male]; Femur: 14–19 years [female], 16–20 years [male], after Scheuer and Black 2000), Kurki et al.'s (2022) plots suggest that cortical area may continue to increase in long bone diaphyses following their average time of epiphyseal fusion. In metacarpals specifically, Martin et al. (2010) examined cortical thickness, width, medullary diameter, and length in individuals under 18 years old. They reported that metacarpal cortical thickness and total width measures, reflective of periosteal growth, began to plateau around age 14 in girls but continued to increase in boys until approximately 16–18 years of age (total width at 17–18 years old, thickness at 16 years old) (Martin et al. 2010). Frisancho et al. (1970) also found that significant endosteal apposition began between the ages of 11 and 14 in girls and 17 and 18 in boys, and that the cortical area of both sexes continued to expand until age 30.

Evidence from both human and non‐human animals suggests that diaphyseal cortical bone continues to expand after epiphyseal fusion; however, the specific nature and relationship of this growth as it relates to the timing of epiphyseal fusion of the metacarpal bones is less clear. This study investigates if there are significant increases in the amount of bone accrued post‐fusion, as this continued appositional growth has the potential to impact the reliability of cortex‐based quantitative analyses in the youngest fully fused individuals; if these individuals are still acquiring bone, they may not be well suited for radiogrammetric comparison against other fully mature adults. In this study, we utilized a documented, known‐age and sex sample of longitudinal hand‐wrist radiographs from individuals between 16 and 20 years old to clarify if and how cortical bone apposition continues in the second and third metacarpals following epiphyseal fusion. This age range spans the transition period between the middle adolescent (15–17 years) and late adolescent (18–24) age categories (as defined by Lewis 2022), and includes the age at which Martin et al. (2010) found metacarpal bone acquisition to level off (18 years). Using this sample, we aim to clarify: (1) if and until which age cortical bone properties continue to change, and (2) if those changes are located predominantly on the periosteal and/or endosteal surface(s). Based on published human and animal models, we hypothesize that fused metacarpals continue to accrue cortical bone width during the mid‐to‐late adolescent transition (i.e., between 16 and 20 years old). By improving the understanding of metacarpal cortical bone growth following fusion, we aim to support biological anthropologists and archaeologists studying adolescence, growth and development, and later‐life bone loss in the selection and interpretation of metacarpal bone samples for radiogrammetric comparison.

## 2 Materials and Methods

This study investigates the second and third metacarpals (MC2 and MC3) in hand‐wrist radiographs from the Burlington Growth Study (BGS) (1952–1971) (Thompson and Popovich 1977). As part of the BGS, annual hand‐wrist radiographs of white, adequately nourished, high socioeconomic status, suburban children between the ages of 3 and 21 were collected (Suri et al. 2013; Thompson and Popovich 1977). The BGS included approximately 85%–90% of all the children (within the study's age ranges) living in Burlington at this time, and was considered representative of the city's population and not biased based on health or developmental status (Suri et al. 2013; Thompson and Popovich 1977). The BGS sub‐sample used in the current study consists of de‐identified and digitized anteroposterior hand‐wrist radiographs (Burlington Growth Centre 2017). As not all individuals returned annually for follow‐up radiographs, the sample size for this study was maximized by selecting a biennial sample beginning at age 16 (the age at which most MC2s are fully fused). A total of 54 individuals (female, *n* = 28; male, *n* = 26) with fully fused metacarpals, clear diaphyseal endosteal borders, and recorded sex met the inclusion criteria. All 54 individuals underwent X‐ray imaging at the age of 16; 43 were X‐rayed again at 18 years old and 30 returned for radiographs at 20 years old. A total of 19 people had X‐ray images at all three ages (16, 18, and 20) (Table 1). Very few individuals returned for X‐ray imaging at 21 years, so this age was excluded from the current study.

**TABLE 1:** Number of individuals with radiographs at each age, alongside the number of individuals who returned for radiographs at the ages of only 16 and 18, only 16 and 20, and all ages (16, 18, and 20 years old). Individuals were included in counts if they had at least one comparative age present (e.g., 16 year old individuals who did not return for radiographs at 18 or 20 years old were excluded from the study).

Sex	*n* individuals with X‐rays at each age			*n* compared individuals by age^a^			Total *n* of individuals
	*n* 16 years	*n* 18 years	*n* 20 years	*n* individuals X‐rayed at only ages 16 and 18	*n* individuals X‐rayed at only ages 16 and 20	*n* individuals X‐rayed at all ages (16, 18, and 20 years old)	
Male	26	20	15	11	6	9	26
Female	28	23	15	13	5	10	28
Total	54	43	30	24	11	19	54

^a^ Sample counts included individuals with radiographs at all ages (16, 18, and 20 years old), as well as those with radiographs at 16 and either only at 18 or only at 20. For example, a statistical test that compared males aged 16 with those aged 18 included the 11 individuals with radiographs at only those two ages, plus the nine individuals with radiographs at all three ages, for a total sample size of 20.

The film radiographs were digitally scanned as TIFF image files of the same pixel density (300 pixels per inch [PPI]) by the Burlington Growth Centre and curated by the American Association of Orthodontists Foundation Craniofacial Growth Legacy Collection (AAOF) (AAOF Legacy Collection 2022). As the hand‐wrist radiographs lacked a known‐size reference for calibration to absolute units, the measurements in this study were recorded in pixels and measured values were directly compared only within this particular study sample. As an additional precaution, author MS measured and compared the radiopaque lead markers on each image in order to identify any possible magnification differences related to variation in X‐ray technique and to further ensure the comparability of the measurements taken from these radiographic images; the lead markers were found to be the same dimensions across all images, suggesting the radiographs were obtained using comparable technique and magnification.

**FIGURE 1:**
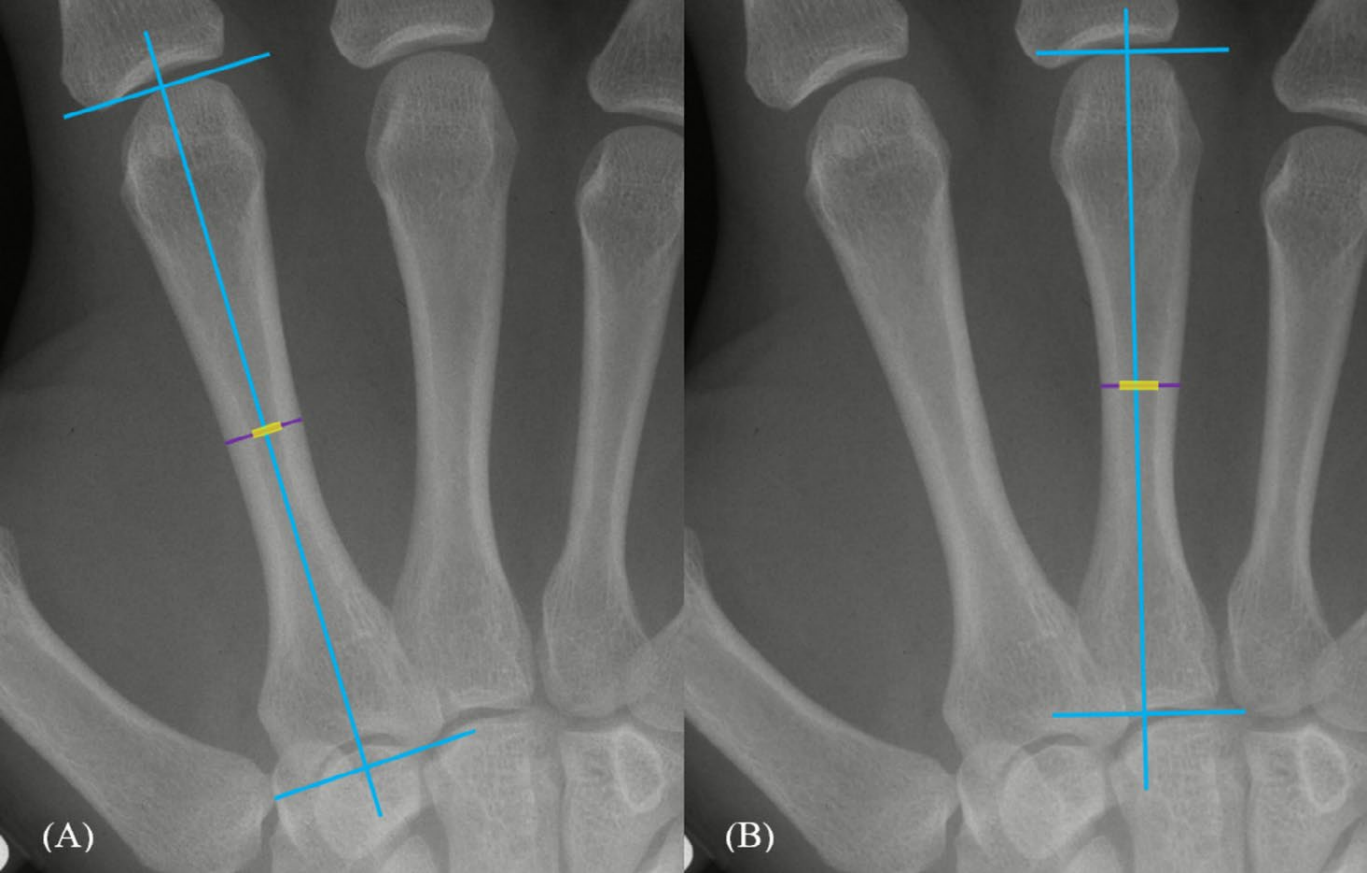
Placement of metacarpal total length and width measurements for the MC2 (A) and MC3 (B). Blue lines represent guides used to find the total lengths and perpendicular midpoints. The dark purple line indicates the total width at the midpoint; the yellow line indicates the medullary width at the midpoint, measured along the same line as the total width.

The total length, total width (TW), and medullary width (MW) of every MC2 and MC3 were measured on digital X‐ray images using ImageJ (NIH) v. 1.53 software. All measures reported in this study were taken by MS; detailed instructions on method, measurement, and the calculation of inter‐ and intra‐observer error are described and reported in Schneider and Gilmour (2023). Total MC2 and MC3 lengths were measured from the distal‐most projection of the metacarpal head to the proximal‐most aspect of the trapezoid facet (MC2) and the most visible proximal point, excluding the styloid process due to radiographic overlap (MC3, see also Harris et al. 1992) (Figure 1). TW and MW were measured at the metacarpal midpoints following detailed guidelines outlined in Schneider and Gilmour (2023). The cortical bone's endosteal border was defined following Meema and Meema (1987) and Ives and Brickley (2004). TW and MW were used to calculate the cortical index (CI), a cortical bone thickness ratio standardized by the metacarpal's diameter (TW) in order to account for variation in body size:

$$\text{Cortical index}\;\left(\mathrm{CI}\right)\%=\frac{\text{total width}-\text{medullary width}}{\text{total width}}\times 100$$

TW and MW measures in pixels allow for the identification of trends or comparison of measures within this study, whereas the CI is a percentage and therefore a dimensionless ratio that can be directly compared to other studies.

Data were analyzed using SPSS (version 29). All data (MW, TW, CI) for both sexes and all three age groups were analyzed for normality using a Shapiro–Wilks test (Supporting Information A). As some variables were not normally distributed and some sample sizes were relatively small, non‐parametric tests were selected for all inferential statistical comparisons. Unless otherwise stated, results were considered significant at a *p*‐value of ≤ 0.05. Sex differences within CI, MW, and TW were examined separately for each age category using Mann–Whitney *U* tests. Wilcoxon signed‐rank tests were used to compare within‐individual, age‐related MW, TW, and CI changes; within‐individual changes were considered significant at an adjusted *p*‐value of ≤ 0.017 (Bonferroni multiple‐comparison correction of *p* ≤ 0.05). The number of individuals with radiographs that could be compared between each set of ages is reported in Table 1. To better understand the size and magnitude of differences present, effect sizes (*r*) were calculated for the Wilcoxon signed‐rank test results by dividing the *z*‐statistic by the square root of the number of samples (*n*); effect size differences between groups were interpreted as large (*r* = 0.5), medium (*r* = 0.3), small (*r* = 0.1), or no effect/very small (*r* < 0.1) (Rosenthal 1991).

## 3 Results

CI, MW, and TW of both MC2 and MC3 were compared between males and females at ages 16, 18, and 20 (Tables 2 and 3, sample sizes reported in Table 1). MW and TW differed significantly between the sexes for both MC2 and MC3 and at every age, whereas CIs were not significantly different between females and males at any age. These results can be explained by the effect of sex‐related body size differences; CI measures were likely comparable between males and females because the CI equation controls for the effect of body size and robusticity. Due to their similarity, male and female CI data were combined for subsequent statistical comparisons, whereas all MW and TW data were analyzed separately by sex.

**TABLE 2:** Cortical index (CI), total width (TW), and medullary width (MW) means and standard deviations (CI units reported in percentages, MW and TW units in pixels) for both sexes, MC2 and MC3, and all age categories.^a^

Metacarpal	Sex	CI (%)			MW			TW		
		16 years	18 years	20 years	16 years	18 years	20 years	16 years	18 years	20 years
MC2	Female	63.0 ± 6.1	65.0 ± 6.7	63.5 ± 5.9	33.3 ± 6.5	31.9 ± 6.6	33.4 ± 7.3	89.8 ± 6.3	90.8 ± 5.5	90.7 ± 7.6
	Male	60.7 ± 7.0	61.8 ± 7.3	60.4 ± 8.4	42.5 ± 9.1	42.6 ± 9.7	45.4 ± 10.8	107.3 ± 7.8	110.4 ± 7.1	114.0 ± 6.2
	Sex combined	61.9 ± 6.6	63.5 ± 7.1	62.0 ± 7.4	37.7 ± 9.1	36.9 ± 9.8	39.4 ± 11.0	98.2 ± 11.3	99.9 ± 11.6	102.4 ± 13.5
MC3	Female	58.3 ± 7.4	59.2 ± 7.8	58.4 ± 7.5	37.2 ± 8.0	37.1 ± 8.7	38.1 ± 8.4	89.0 ± 7.7	90.5 ± 8.2	91.3 ± 6.8
	Male	56.1 ± 8.7	58.7 ± 8.2	57.8 ± 8.6	46.0 ± 11.6	44.6 ± 11.8	46.6 ± 11.2	103.5 ± 8.7	106.6 ± 9.4	109.4 ± 6.3
	Sex combined	57.3 ± 8.1	59.0 ± 8.0	58.1 ± 8.1	41.4 ± 10.8	40.6 ± 10.9	42.4 ± 10.7	96.0 ± 10.9	98.0 ± 11.9	100.3 ± 11.2

^a^ MC2 = second metacarpal; MC3 = third metacarpal.

**TABLE 3:** Sex differences in cortical index (CI), total width (TW), and medullary width (MW) measures at each age (16, 18, and 20 years old) tested using Mann–Whitney *U*.^a^

Metacarpal	CI			MW			TW		
	16 years	18 years	20 years	16 years	18 years	20 years	16 years	18 years	20 years
MC2	*U* = 259.000, *p* = 0.069	*U* = 150.000, *p* = 0.051	*U* = 88.000, *p* = 0.310	***U* = 147.000, *p* < 0.001**	***U* = 76.000, *p* < 0.001**	***U* = 36.500, *p* = 0.002**	***U* = 29.000, *p* < 0.001**	***U* = 8.500, *p* < 0.001**	***U* = 1.000, *p* < 0.001**
MC3	*U* = 289.000, *p* = 0.194	*U* = 220.000, *p* = 0.808	*U* = 102.000, *p* = 0.663	***U* = 180.500, *p* = 0.001**	***U* = 135.500, *p* = 0.021**	***U* = 52.000, *p* = 0.012**	***U* = 86.500, *p* < 0.001**	***U* = 52.000, *p* < 0.001**	***U* = 10.000, *p* < 0.001**

^a^ Bolded values indicate significant differences. MC2 = second metacarpal; MC3 = third metacarpal.

Regardless of sex, MC2 and MC3 CI and TW clearly increased within individuals until at least age 18, while MW remained relatively unchanged over time (Table 4). Medium‐sized CI increases were identified between ages 16 and 18 for both MC2 and MC3. Additionally, between the ages of 16 and 20 years, a large and significant increase in MC3 CI was observed (*Z* = −2.890, *p = 0.004*, *r* = 0.53). For both MC2 and MC3, TW showed large and significant differences between the ages of 16 and 18, and 16 and 20, except for female MC2s, where no significant difference was present between the ages of 16 and 18. TW comparisons between the older ages of 18 and 20 did not reach significance, but they did demonstrate medium‐ to large‐sized differences generally indicative of a continued increase in metacarpal TW, likely explained by periosteal apposition. In contrast to CI and TW, MW did not significantly change with age; MC2s of females between 16 and 18 years old are again the exception, as they exhibited a significant decrease in MW at the age of 18, suggestive of medullary canal narrowing that may be explained by endosteal apposition.

**TABLE 4:** Age differences in cortical indices (CI), total widths (TW), and medullary widths (MW) between male and female individuals 16, 18, and 20 years old, compared using Wilcoxon signed‐rank tests and effect sizes (*r*).^a^

Ages	MC2					MC3				
	CI	Males		Females		CI	Males		Females	
		MW	TW	MW	TW		MW	TW	MW	TW
16 vs. 18	***Z* =** −**2.946, *p* = 0.003, *r* =** −**0.449**	*Z* = −0.112, *p* = 0.911, *r* = −0.025	***Z* =** −**3.493, *p* < 0.001, *r* =** −**0.781**	***Z* =** −**3.366, *p* < 0.001, *r* =** −**0.702**	*Z* = −1.348, *p* = 0.178, *r* = −0.281	***Z* =** −**2.451, *p* = 0.014, *r* = −0.373**	*Z* = −1.401, *p* = 0.161, *r* = −0.313	***Z* =** −**3.764**, ***p* < 0.001**, ***r* = −0.842**	*Z* = −0.487, *p* = 0.627, *r* = −0.102	***Z* =** −**2.718, *p* = 0.007**, ***r* =** −**0.578**
18 vs. 20	*Z* = −1.610, *p* = 0.107, *r* = −0.369	*Z* = −0.653, *p* = 0.514, *r* = −0.218	*Z* = −1.684, *p* = 0.092, *r* = −0.561	*Z* = −1.685, *p* = 0.092, *r* = −0.533	*Z* = −1.266, *p* = 0.205, *r* = −0.400	*Z* = −1.610, *p* = 0.107, *r* = 0.369	*Z* = −0.178, *p* = 0.858, *r* = −0.059	*Z* = −0.952, *p* = 0.341, *r* = −0.317	*Z* = −1.840, *p* = 0.066, *r* = −0.582	*Z* = −1.723, *p* = 0.085, *r* = −0.545
16 vs. 20	*Z* = −1.697, *p* = 0.090, *r* = −0.310	*Z* = −0.142, *p* = 0.887, *r* = −0.037	***Z* =** −**3.041**, ***p* = 0.002**, ***r* = −0.785**	*Z* = −1.445, *p* = 0.148, *r* = −0.373	***Z* =** −**2.855**, ***p* = 0.004**, ***r* = −0.737**	***Z* =** −**2.890**, ***p* = 0.004**, ***r* = −0.528**	*Z* = −1.782, *p* = 0.075, *r* = −0.460	***Z* =** −**3.128, *p* = 0.002, *r* = −0.808**	*Z* = −0.967, *p* = 0.334, *r* = −0.250	***Z* =** −**3.021, *p* = 0.003, *r* = −0.780**

^a^ Bolded values indicate significant differences after the application of the Bonferroni multiple comparison correction, after which *p*‐values were considered significant at ≤ 0.017. Effect sizes (r) of ≥ 0.5 were considered large. MC2 = second metacarpal; MC3 = third metacarpal.

In this sample, the results suggest that the CI continues to increase notably until at least 18 years of age, and perhaps to a lesser degree, until 20 years of age or older. The TW findings indicate a continued periosteal widening of the metacarpal diaphyses between the ages of 16 and 20, with a reduction in the magnitude of this change between the ages of 18 and 20. The MW tends to stay relatively stable between the ages of 16 and 20. However, cortex changes in female MC2s between the ages of 16 and 18 stand out as slightly different from the MC3 and male measures in that they exhibit reduced periosteal apposition (as indicated by TW), but increased endosteal apposition (indicated by MW).

## 4 Discussion

Despite complete epiphyseal fusion of these metacarpal bones, our research identified discernible changes in cortical bone indicative of clear and continued cortical modeling along the diaphysis until approximately 18 years of age. Over the four years of growth covered in this study, we observed the greatest and most significant cortical changes in TW and CI between the ages of 16 and 18 years old. Specifically, the CI and TW of both MC2 and MC3 increased, whereas the MW remained relatively stable, a pattern suggestive of bone apposition at the periosteal surface with minimal apposition or resorption at the endosteal margin. During growth phases, an increasing CI is generally attributed to periosteal bone apposition paired with slower endosteal resorption (Bass et al. 1999; Chevalley and Rizzoli 2022; Garn 1970; Henry et al. 2004). Our 16 to 18 year old metacarpal‐specific findings underscore this process and align with results reported by Martin et al. (2010), who suggested that periosteal and endosteal expansion in the metacarpals slows, and cortical bone growth largely plateaus by 18 years of age in males and 17 years in females.

Between the ages of 18 and 20 years, TW stabilized with slight, non‐significant increases in MW and decreases in CI. Preliminarily, these results may indicate medullary canal expansion caused by endosteal resorption, a pattern that would also explain the slightly smaller average CI measures at 20 years old. However, metacarpal CI is typically documented to continue to increase until 30 years of age (Böttcher et al. 2006; Shepherd et al. 2005), corresponding with a slight increase in mass throughout the skeleton in the third decade of life (Baxter‐Jones et al. 2011). The decrease in CI and increase in MW between ages 18 and 20 were therefore unexpected and should be interpreted cautiously, especially given the smaller sample size available for 18 and 20 year old comparisons. Participant attrition in longitudinal studies is a known issue (Tambs et al. 2009; Gustavson et al. 2012), and this reduction in BGS participants who returned at both ages 18 and 20 is not surprising. Within the BGS, there are other individuals between these ages who were excluded from the current study because they did not have radiographs at 16 years old, a key criterion for inclusion in this research about bone acquisition immediately following metacarpal fusion. Future studies should consider exploring the nature of cortical bone change among these older BGS individuals, as well as within other longitudinal studies in other regions.

Although the trend identified in this study suggests cortical bone apposition at the subperiosteal margin plateaus around the age of 18 for both sexes, our data indicated a pattern that is potentially indicative of different growth trajectories between the sexes and metacarpals. Namely, the female MC2s between 16 and 18 years old did not adhere to the general trend of increasing TW and CI and stable MW. Instead, for this specific element and sex, expansion was not occurring at the periosteal margin of this bone as expected, but rather on the endosteal surface. This pattern aligns with Garn's (1970) observation that, post‐puberty, cortical thickening in males is mainly periosteal, while in females it involves both periosteal and endosteal apposition. Earlier skeletal maturation in females, linked to the adolescent growth spurt and pubertal hormones (Sanders et al. 2017; Scheuer and Black 2000; Tanner et al. 1976; Bass et al. 2002), may explain the earlier plateauing of MC2 dimensions in girls, as also noted by Martin et al. (2010). In the metacarpal specifically, Martin et al. (2010) note that growth in the element length, TW, and MW levels off earlier in girls than in boys. Endosteal appositional trends are specifically described by Frisancho et al. (1970) based on samples of children from the USA (Ohio) and Central America. Frisancho et al. (1970) reported a shift from endosteal resorption to apposition at approximately 12 years in females and 16 years in males, with endosteal deposition accounting for a larger portion of cortical gains in females (36%) than in males (23%). If our Burlington sample reflects this resorption‐apposition shift, it appears to occur later than in the Ohio children studied by Frisancho et al. (1970), but aligns with findings in other groups, such as high‐altitude Peruvian populations, where endosteal apposition began around ages 16–18 (Frisancho et al. 1970). This shift may occur around puberty, with estrogen acting to suppress periosteal growth and enhance endosteal apposition (Garn 1970). However, Frisancho et al. (1970) believed that more than sexual maturation was involved, emphasizing that environmental factors could contribute to endosteal differences and the timing of the appositional shift. The endosteal apposition was only observed in the Burlington female MC2s, which may be explained by the relative delay in male endosteal apposition noted by Frisancho et al. (1970). As such, it is plausible that this endosteal transitional phase is not clearly captured for all individuals in the current study, given that the majority of final radiographs were taken at a maximum age of 20 years. Endosteal appositional trends in individuals up to and over 20 years would provide an interesting avenue of inquiry for future studies.

In order to continue to describe these changes, following fusion, future studies would benefit from investigations of different populations and time periods, as they relate to additional factors that influence cortical bone growth (e.g., hormones, nutrition, physical activity, weight, age at menarche, and environment) (Gordon et al. 2017; Sanders et al. 2017; Chevalley and Rizzoli 2022). For example, external lifestyle factors, such as diet or exercise, are estimated to account for 20%–40% of adult peak bone mass attainment (Weaver et al. 2016). Similarly, reduced activity could result in adult bone loss at the endosteal margin (Schlecht et al. 2012). Due to the de‐identified nature of the sample, data on these lifestyle variables were unavailable and could not be controlled for, but it is possible that they affected cortical bone modeling at this age in this sample. Future consideration of these interacting factors, alongside contemporary age categories of adolescence: early adolescence (10–14 years), middle adolescence (15–17 years), and late adolescence (18–24 years) (e.g., Lewis 2022), may also help align cortical bone studies with contemporary work on the adolescence‐adult transition. Including individuals as young as 10 and as old as 30 would build on the results presented in this study and those of previous scholars, such as Frisancho et al. (1970), allowing for a more comprehensive understanding of bone development throughout the whole span of adolescence and into young adulthood.

In summary, this study aimed to (1) clarify if, and until what age, appositional growth occurs in the cortical bone of metacarpal diaphyses, and (2) identify whether these cortical changes occur primarily at the endosteal or periosteal margin(s). This study found that following epiphyseal fusion and until approximately age 18, the MC2 and MC3 of both males and females continued to accrue cortical bone, predominantly at the periosteal surface. Although preliminary, these findings help inform the selection of comparative samples for metacarpal radiogrammetry research. Namely, even if fully fused, the metacarpals of individuals younger than 18 years old are still accruing significant amounts of cortical bone and therefore should not be included in comparative peak bone mass samples. While cortical changes slow at 18 years of age, caution should also be used when considering the inclusion of individuals between the ages of 18 and 20 years old. Given that significant cortical bone changes appear to slow or cease by age 20, we recommend selecting individuals over the age of 20 for adult metacarpal radiogrammetry analyses.

## Author Contributions

**Maris A. Schneider:** conceptualization, methodology, investigation, formal analysis, visualization, writing – original draft, writing – review and editing. **Rebecca J. Gilmour:** conceptualization, methodology, data curation, investigation, supervision, project administration, writing – original draft, writing – review and editing.

## Funding

Radiographs were made available to the researchers at no cost thanks to the Craniofacial Growth Legacy Collection and the Burlington Growth Centre.

## Ethics Statement

Ethics approval was granted for this study by the Mount Royal University Human Research Ethics Board (Application Number 102670).

## Conflicts of Interest

The authors declare no conflicts of interest.

## Supporting information


**Supporting Information:** Shapiro–Wilks test results for data normality by metacarpal element, sex, and age.

## Data Availability

The authors of the article are not authorized to make individual datasets publicly available, but data can be requested from the Burlington Growth Centre.
