# Supplementary material for: Continued Metacarpal Cortical Bone Growth in Mid to Late Adolescence: A Longitudinal Study of Cortical Bone Acquisition in a Documented Sample of 16‐ to 20‐Year‐Olds
Source: Am J Biol Anthropol. 2025 Dec 18;188(4):e70186. doi: 10.1002/ajpa.70186 (PMC12712780; doi:10.1002/ajpa.70186)
Supplement: Supplementary file 1 — Supporting Information: Shapiro–Wilks test results for data normality by metacarpal element, sex, and age. [file AJPA-188-e70186-s001.docx]

**Supporting Information**

Shapiro-Wilks test results for data normality by metacarpal element, sex, and age.

| **Element** | **Measure** | **Both Sexes** | | | | **Female** | | | **Male** | | |
| --- | --- | --- | --- | --- | --- | --- | --- | --- | --- | --- | --- |
|  |  | **All ages** | **16 years old** | **18 years old** | **20 years old** | **16 years old** | **18 years old** | **20 years old** | **16 years old** | **18 years old** | **20 years old** |
| MC2 | MW | W(127)=.993, p=.740 | W(54)=.981, p=.538 | W(43)=.989, p=.952 | W(30)=.984, p=.925 | W(28)=.962, p=.383 | W(23)=.960, p=.464 | W(15)=.983, p=.987 | W(26)=.933, p=.092 | W(20)=.932, p=.168 | W(15)=.959, p=.682 |
|  | TW | **W(127)=.967, p=.003** | W(54)=.970, p=.189 | **W(43)=.943, p=.034** | W(30)=.958, p=.273 | W(28)=.967, p=.511 | W(23)=.969, p=.672 | W(15)=.962, p=.726 | W(26)=.958, p=.357 | W(20)=.948, p=.337 | W(15)=.961, p=.715 |
|  | CI | W(127)=.998, p=.320 | W(54)=.980, p=.500 | W(43)=.982, p=.720 | W(30)=.987, p=.967 | W(28)=.953, p=.229 | W(23)=.976, p=.821 | W(15)=.974, p=.909 | W(26)=.923, p=.052 | **W(20)=.892, p=.030** | W(15)=.956, p=.624 |
| MC3 | MW | **W(127)=.972, p=.009** | W(54)=.960, p=.072 | W(43)=.970, p=.318 | W(30)=.954, p=.213 | **W(28)=.910, p=.019** | W(23)=.968, p=.633 | W(15)=.898, p=.090 | **W(26)=.920, p=.045** | W(20)=.951, p=.388 | W(15)=.888, p=.063 |
|  | TW | **W(127)=.957, p<.001** | W(54)=.958, p=.056 | **W(43)=.945, p=.038** | W(30)=.942, p=.101 | W(28)=.940, p=.108 | W(23)=.925, p=.087 | W(15)=.988, p=.998 | W(26)=.946, p=.186 | W(20)=.936, p=.203 | **W(15)=.879, p=.046** |
|  | CI | **W(127)=.966, p=.003** | W(54)=.964, p=.105 | W(43)=.965, p=.205 | W(30)=.951, p=.185 | W(28)=.936, p=.087 | W(23)=.964, p=.555 | W(15)=.925, p=.229 | **W(26)=.885, p=.007** | W(20)=.946, p=.317 | **W(15)=.879, p=.046** |

Bolded values indicate significant differences. CI = Cortical Index, MW = Medullary Width; TW = Total Width; MC2 = Second Metacarpal; MC3 = Third Metacarpal.
